# Enhanced Skin Incisional Wound Healing With Intracellular ATP Delivery *via* Macrophage Proliferation and Direct Collagen Production

**DOI:** 10.3389/fphar.2021.594586

**Published:** 2021-06-16

**Authors:** Harshini Sarojini, Alexander Bajorek, Rong Wan, Jianpu Wang, Qunwei Zhang, Adrian T. Billeter, Sufan Chien

**Affiliations:** ^1^ Department of Surgery, School of Medicine, University of Louisville, Louisville, KY, United States; ^2^ Department of Environmental and Occupational Health Sciences, School of Public Health and Information Sciences, University of Louisville, Louisville, KY, United States

**Keywords:** wounds, dehiscence, incisional, healing, tensile strength, ATP, macrophage, collagen

## Abstract

This study sought to use a newly developed intracellular ATP delivery to enhance incisional wound healing to reduce surgical wound dehiscence and to explore possible mechanism for this effect. Thirty-five adult New Zealand white rabbits were used. Skin incisions were made on the back and closed. ATP-vesicles were mixed with a neutral cream for one side of the wounds while the neutral cream alone was used on the other side of the wounds. Laser speckle contrast imaging (LSCI), biomechanical, histological, and immunohistochemical analyses were performed 7 and 14 days after surgery, and macrophage culture was used to test the enhanced collagen production ability. Among them, 10 were used for wound perfusion study and 25 were used for wound biomechanical and histological/immunohistochemical studies. Wound tissue perfusion was reduced after surgery especially in early days. Wound tissue tensile strength, breaking stress, and elasticity were all much higher in the ATP-vesicle treated group than in the cream treated group at days 7 and 14. The healing was complemented by earlier macrophage accumulation, *in situ* proliferation, followed by direct collagen production. The results were further confirmed by human macrophage culture. It was concluded that intracellular ATP delivery enhanced healing strength of incisional wounds *via* multiple mechanisms.

## Introduction

An estimated 312.9 million surgical procedures were undertaken worldwide (Weiser et al., 2015). A patient’s recovery and rehabilitation depends mainly on the postoperative wound healing processes. Surgical wound dehiscence (SWD) impacts on mortality and morbidity rates of patients, considerably contributing to prolonged hospital stays (Sandy-Hodgetts et al., 2013). SWD, is defined as partial or total disruption of any or all layers of the operative wounds (Moossa et al., 1997; Petersson et al., 2014), and the occurrence rate varies dramatically from 0.25% to as high as 42.3% (Scalise et al., 2016). The type of breakdown ranges from simple skin dehiscence and hernia formation to the most severe and potentially lethal forms of breakdown characterized by evisceration, gastrointestinal anastomotic leaks, pancreatic fistulas, and vascular pseudo-aneurysms (Scalise et al., 2016). Wound dressings provide a physical barrier between the injury site and outside environment, inhibiting further damage or infection therefore they are important for wound healing. Wound dressings manage and encourage the wound healing procedure for proper recovery (Aduba and Yang, 2017). To compare with excisional wounds, there have been few studies exploring dressing effect on wound healing strength, such as egg white/polyvinyl alcohol/clay nanocompositve hydrogel (Sirousazar et al., 2016), poly(vinyl alcohol)/clay nanocomposite hydrogel (Sirousazar et al., 2011), connective tissue growth factor/CCN2 (Henshaw et al., 2015), hydrocolloid dressing (Quirinia and Viidik, 2001), 3M’s Micropore (Hever et al., 2019), mupirocin dressing (Ahmad et al., 2019), dialkylcarbamoylchloride-coated dressings (Totty et al., 2019), and a combination of vitamin E and silicone (E-Sil) (Ruiz-Tovar et al., 2019) but so far no dressing has shown consistent effect. In recent years, negative pressure wound therapy (NPWT) has gained increased attention because it has shown more positive effects in many reports (Sahabally et al., 2018; Hyldig et al., 2019; Svensson-Bjork et al., 2019). Even so, the recent largest case studies in the Cochrane review conclude that it is “low or very low certainty for all outcomes” (Webster et al., 2019; Norman et al., 2020). This is understandable because the pathophysiology of SWD is complex. It is well-known that multiple factors contribute to SWD (Hahler 2006), and many of the pathways involved are still not entirely clear. Malnutrition, sepsis, anemia, uremia, liver failure, diabetes, obesity, malignancy, use of steroids, and many other diseases can all contribute to SWD (Aduba and Yang, 2017). It is a basic clinical observation that wounds do not heal in tissue that does not bleed, whereas they always heal in tissue that bleeds extensively (Niinikoski, 2002; Gottrup, 2004). Surgeons have long recognized that ischemic tissues are easily infected and heal poorly, the critical ischemic nature of surgical wounds has not gotten adequate attention (Franz et al., 2007). One direct effect of ischemia is a reduction of tissue high energy phosphate reserve. In previous studies we reported positive effect of intracellular delivery of Mg-ATP (ATP-vesicles) to enhance excisional skin wound healing process in non-ischemic and ischemic wounds both in non-diabetic and diabetic animals (Chien, 2006; Wang et al., 2009a; Wang et al., 2010; Chien, 2010a; Chien, 2010b; Howard et al., 2014; Sarojini et al., 2017; Mo et al., 2020). One unique feature of intracellular delivery of ATP-vesicles was the very early and massive macrophage accumulation, and these studies provide the primary evidence of *in situ* macrophage proliferation, M2 polarization, and direct collagen production during wound healing (Chien, 2006; Wang et al., 2010; Chien, 2010a; Chien, 2010b; Sarojini et al., 2017; Mo et al., 2020) without the involvement of other known factors such as parasite infection (Jenkins et al., 2011). This feature could be especially valuable for the healing of incisional wounds because the healing of incisional wounds depends more heavily on the formation of the extracellular matrix, especially tissue collagen content (Klinge et al., 2006; Chang et al., 2010; Petersson et al., 2014). We hypothesized that the early and massive macrophage accumulation, proliferation, M2 polarization, and direct collagen production could enhance incisional wound healing. This study aims to assess the effects of intracellular delivery of Mg-ATP (ATP-vesicles) on histology and tensiometrical properties in the healing of incisional wounds in rabbits and provides a preliminary exploration of the possible mechanisms.

## Materials and Methods

### ATP-Vesicles and Their Basic Performance Studies

The ATP-vesicles were provided to us in a freeze-dried form from Avanti Polar Lipids Inc. (Alabaster, AL). They were stored at -20°C and were reconstituted with a neutral cream (Velvachol) prior to use. After reconstitution, the composition was: 100 mg/ml of Soy Phosphatidylcholine (Soy PC)/1,2-dioleyl-3-trimethylammonium propane (DOTAP) (50:1), Trehalose/Soy PC (2:1), 10 mM KH_2_PO_4_, and 10 mM Mg-ATP. The lipid vesicles diameters ranged from 120 to 160 nm. For simplicity, we will use “ATP” to represent Mg-ATP and use “ATP-vesicles” to represent the Mg-ATP-encapsulated small diameter lipid vesicles in this article.

Before launching this study, we characterized the effects of ATP-vesicles in the following aspects:

1. Cell penetration. We used cell culture to confirm its ability to fuse with cell membrane and deliver the content into the cytosol (Chien, 2006; Chien, 2010a; Chien, 2010b).
2. Cell protection. We used human umbilical vein endothelial cells (HUVECs) and cardiomyocytes to compare ATP-vesicles with their individual components (Mg-ATP alone, lipid vesicles alone, and Mg-ATP plus lipid vesicles) and confirmed that the protective effects on cell survival during extreme hypoxia (<0.5% O_2_)/chemical hypoxia (induced by KCN) were only obtained with ATP-vesicles (Chien, 2006; Chien, 2010a; Chien, 2010b).
3. Stability test. We stored the ATP-vesicles in freezer for more than 3 years, and subjected the preparation to 10 cycles of freezing-thawing-sonication at room temperature, NMR spectroscopy did not show any degradation of Mg-ATP. This feature makes it suitable for wound dressing.
4. Skin penetration. We used rat and pig skins for penetration study and found 10–20 folds increase of skin penetration rate with ATP-vesicles when compared to free Mg-ATP (Chien, 2010a).
5. Dose-response in wound treatment. We found 5–20 mM concentrations of Mg-ATP in the ATP-vesicles have significant results when compared with the dosages above or below this range in wound healing studies.
6. Comparison of ATP-vesicles with their individual components in wound healing. We also compared ATP-vesicles with their two major components–the carrier (empty vesicles) and free Mg-ATP in wound treatment. Neither the empty lipid vesicles nor free Mg-ATP had the same effects as that of ATP-vesicles (Chiang et al., 2007; Chien 2010b).

### Animals and Wounds

All experiments were conducted in accordance with the National Institutes of Health guidelines for the care and use of animals in research, and the protocol was approved by the Institutional Animal Care and Use Committee of the University of Louisville, an AAALAC accredited program (IACUC11030-17153). Prior to the initiation of the study sample sizes and comparisons were determined. The incisional wound study utilized a total of 35 young adult New Zealand white rabbits (2.5–3.0 kg, Myrtle’s Rabbitry, Thompson Station, TN and Charles River Laboratories, Morrisville, NC). All surgery was performed under general anesthesia (ketamine 50 mg/kg and xylazine 5 mg/kg, IM), and all efforts were made to minimize suffering. The back was shaved, sterilized with betadine and alcohol, and draped under aseptic conditions, two 10-cm full thickness skin incisions were made in parallel to the dorsal midline, 30 mm on either side of this line, in the thoracolumbal region. The incisions were made perpendicular to the surface, through the epidermis, dermis, and subcutaneous fascia, but leaving the muscle intact. Bleeding was minimal and easily stopped with gauze pressure. After complete hemostasis, the wounds were closed with 3-0 or 4-0 sutures.

### Postoperative Management

All animals received analgesics postoperatively, including a fentanyl skin patch (25 µg/h) and buprenorphine (0.01 mg/kg, IM) and were permitted free contact to food and water. The experimental wounds were treated with ATP-vesicles (10 mM ATP), reconstituted with normal saline and mixed with a neutral cream (Velvachol, San Antonio, TX, United States), while the control wounds received the neutral cream alone on the other side. The dressing was covered with a piece of cotton paper which in turn was covered with TegaDerm™ (3M, Minneapolis, MN, United States). A nylon jacket prevented scratching of the wound area and dressing removal. Dressing changes were made daily until the sacrifice. Every day old dressings were removed and the wounds were cleaned, microvascular perfusion was measured, photographed, and applied new dressings. The sutures were removed 7 days after surgery. The rabbits were divided into three groups: Sixteen rabbits were used for wound tissue mechanical testing and histological studies—8 of them were euthanized at day 7 and the other eight were euthanized at day 14. The other 19 rabbits were used for wound blood perfusion (10) and immunohistochemical studies (9). In wound perfusion study, a Moor FLPI-2 laser speckle contrast imager (Moor Instruments, Wilmington, DE) was used daily for real-time high-resolution microvascular perfusion measurements. For biochemical and histological analyses, the rabbits were sacrificed at 5, 24, 48 h to 7 and 14 days postoperatively. The wounds, along with surrounding tissues, was removed for analyses.

### Wound Microvascular Perfusion Measurements

The animals were lightly sedated (ketamine 10 mg/kg and xylazine 1 mg/kg, IM), and the hair was shaved. They were resting on a towel and the Moor FLPI-2 connected to a desktop PC was used to scan the wounds and their surrounding areas. The images were recorded and analyzed with Moor FLPI-2 software with high resolution and expressed as flux, a parameter linked to the average speed and concentration of moving red blood cells in the living tissue sample volume (Please see Supplementary Figure S1 for more information).

### Biomechanical Testing

After the wound samples were removed, the skin and wound tissues were soaked in normal saline and tested within 30 min. The tissues were cut into identically sized strips 5 mm wide and 60 mm long with a special metal mold (Please see Supplementary Figure S2 for more information). The strips were cut perpendicularly to the incision, with the incision at the center. For each wound sample, 5–10 skin strips were obtained and the results were pooled to obtain an average value for each wound.

An MTS Insight Material Testing System (MTS Systems, Eden Prairie, MN, United States), with a 1000 N load-cell, was used for tensile strength testing. The skin strip was clamped between two tensile clampers and stretched by moving one clamp at a 6 mm/min crosshead speed until breakage occurred. The load and deformation data were continuously acquired by the MTS Test works four automatic data acquisition system installed on an HP Pentium computer and the following mechanical characteristics were obtained:

The ultimate tensile strength was calculated using the following formula (Sandulache et al., 2003):

Maximum Tensiometer reading (Lbs or Grams) ÷ Cross-sectional area = Tensile strength (MPa or g/mm^2^).

Breaking energy was measured from the load-strain curve as the area between the curve and the *X*-axis to the point of failure (Gottrup, 1983).

Young’s Modulus (psi), also known as tensile modulus is well defined as the ratio of the stress over the strain in the range of stress, is a measure of stiffness of the tissue. It was derived as the slope of the stress–strain curve within the elastic region of the ramp to failure (Gordon et al., 2008).

### Histology and Immunohistochemistry Studies

Histological study was performed by a histologist without the knowledge of dressings used. Samples were fixed in 4% buffered formaldehyde, embedded in paraffin blocks, and 5 µm sections were cut. Hematoxylin and eosin staining was done for general histology evaluation. van Gieson (HT254, Sigma-Aldrich, St. Louis, MO, United States) and picrosirius red (Polysciences, Warrington, PA, United States) staining (Coleman, 2011) was used to estimate the collagen deposition in the wound area. Macrophages were detected by immunohistochemistry staining using anti-MAC387 antibody (AbD Serotec, Raleigh, NC, United States) (Villiers et al., 2006). Histology images were examined with a Nikon Eclipse Ti-E inverted microscope (Nikon Instruments Inc., United States) and different types of collagen stained with picrosirius red were further analyzed by a Zeiss AxioScope circular polarizing microscope (Carl Zeiss, Thornwood, NY, United States). The captured images were further computed morphometrically using Zeiss Axio Imaging software.

### Human Macrophage Culture and Collagen Production

Direct macrophage collagen production in wound healing process is still a controversial issue and many people dismiss this possibility, a cell culture using human macrophage was performed to particularly test the role of macrophages while exclude many confounding *in vivo* factors such as fibroblasts. The procedure for human macrophage primary culture studies was approved by the University of Louisville Institutional Review Board before recruiting any study subjects (HSPPO 08.0018). Written informed consent was obtained from all the study participants. Overall 16 healthy volunteer donors were enrolled; the majority of experiments were piloted with seven donors per experiments. The age of the participants ranged from 19–49 years. Venous blood was collected in EDTA vacutainers (Becton Dickinson, Franklin Lakes, NJ, United States). Primary human monocyte cells were isolated using the magnetic cell sorting method according to the manufacturer’s guidelines. Briefly, the whole blood from the donors was incubated with Human CD14 MicroBeads (Miltenyi Biotec, Auburn, CA, United States) at 37°C in a 5% CO_2_ incubator for 15 min. After washing, the blood was re-suspended in the MACS Separation Buffer (Miltenyi Biotec, Auburn, CA) and run through Whole Blood Columns (Miltenyi Biotec, Auburn, CA). After isolation of cells, the columns were washed three times and the monocytes were eluted from the columns with MACS Elution Buffer (Miltenyi Biotec, Auburn, CA). The isolated cells were washed twice with phosphate buffered saline (PBS) and counted. The purity of the isolated monocytes was >95% as determined by flow cytometry. The primary monocytes were cultured in 1640 RPMI medium (MP Biomedicals, Solon, OH, United States) supplemented with 10% heat-inactivated defined fetal bovine serum, 2 nM L-Glutamine and 100 IU/ml penicillin, 100 µg/ml streptomycin and 250 ng/ml amphotericin B (Thermo Scientific, Waltham, MA, United States). Cells were plated in 24 well culture plates at a concentration of 0.5 × 10^6^ cells/ml/well in humidified incubator with 5% CO_2_ at 37°C.

### Collagen Measurement in Cell Culture

The possibility of direct collagen production by macrophages was tested in primary human macrophages cultures. The cells were treated with ATP vesicles (1 and 10 mM), Mg-ATP (1 mM), lipid vesicles (1 mM) or cream 24 h post plating and for another 24/48 h. The cell culture supernatant was collected and stored at -80°C until further analysis. Collagen type 1-α-1 levels were determined from these culture supernatants through enzyme-linked immunosorbent assays (ELISAs) (My Biosource LLC, San Diego, CA, United States) in 96-well plates according to manufacturer’s protocol. All samples were evaluated in triplicate. Calibration was made using the standard curve generated by the collagen standard provided in the kit.

### Statistical Analyses

Results were reported as mean and standard deviation (SD). The ATP-vesicle treated and cream-treated wounds were equated using commercially available software such as Excel (Microsoft), GraphPad Prism (GraphPad Inc., San Diego, CA, United States), SPSS (IBM), and JMP 10 (SAS Campus Drive, Cary, NC, United States). Our null hypothesis is that there is no difference between the ATP-vesicle treated and cream-treated wounds, thereby generating a *p* value less than 0.05 in all our experiments. Difference between groups was calculated by the Student t-test or ANOVA. A *p* value of <0.05 was considered significant.

## Results

### General Observation

The incisional wounds were created on the back of the rabbits and protected with nylon jacket, no dressing dislodgement occurred and the wounds healed without infection. Gross examination indicated smooth healing lines without edema or separation. At day 7, the healed skin still appeared somewhat fragile in both groups, but the binding was much stronger at day 14. No gross differences were noted between the two groups or different sexes.

### Wound Area Microvascular Perfusion Measurements

**FIGURE 1:**
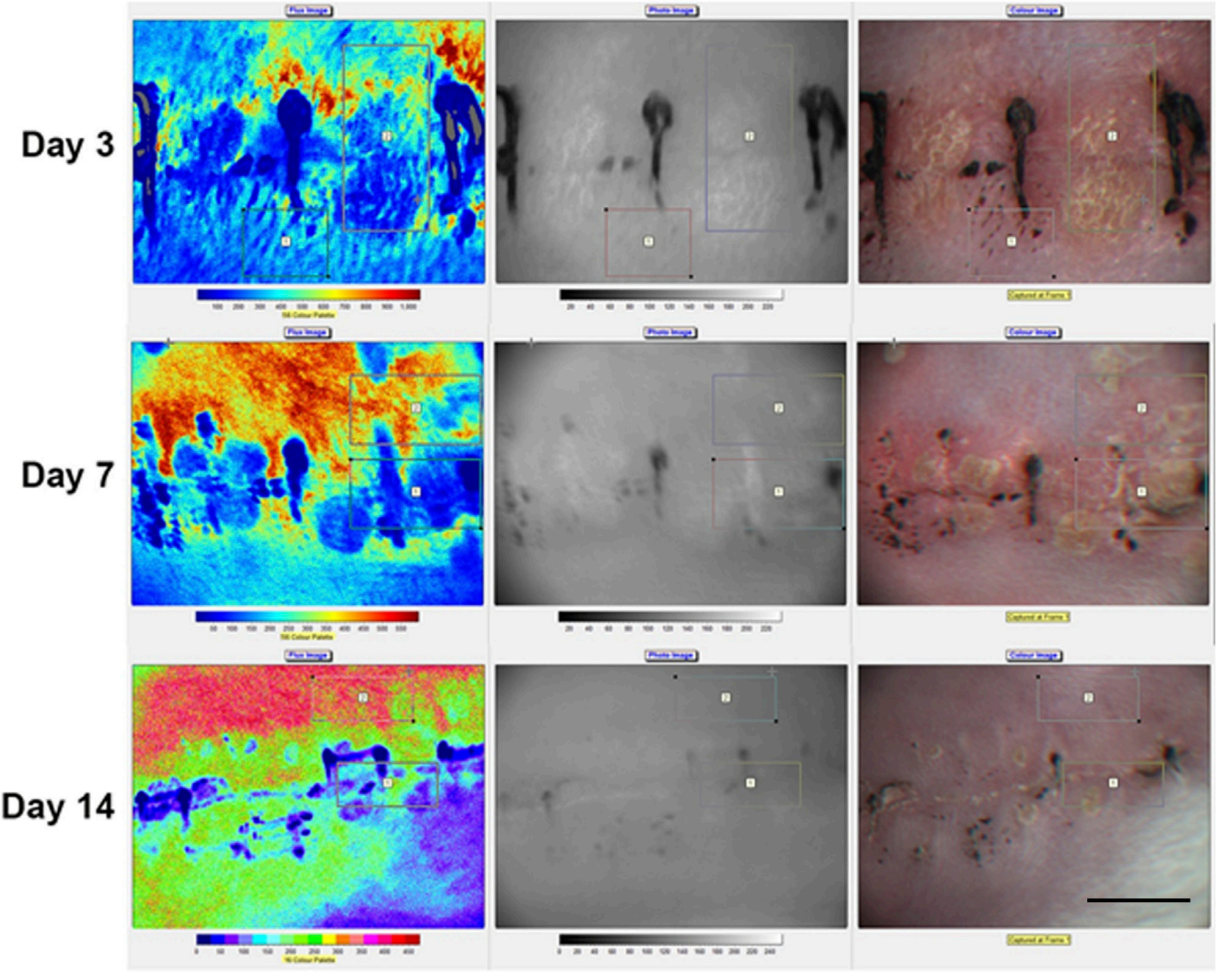
Local blood perfusion in rabbit back skin incisional wounds. By using Moor FLPI-2 full-field Laser Perfusion Imager localized blood perfusion was imaged in the rabbit back skin incisional wounds on days 3, 7 and 14. An increase in blood perfusion can be seen from day 7 onwards (represented by yellow and orange colors). Scale bar is 10 mm.

**FIGURE 2:**
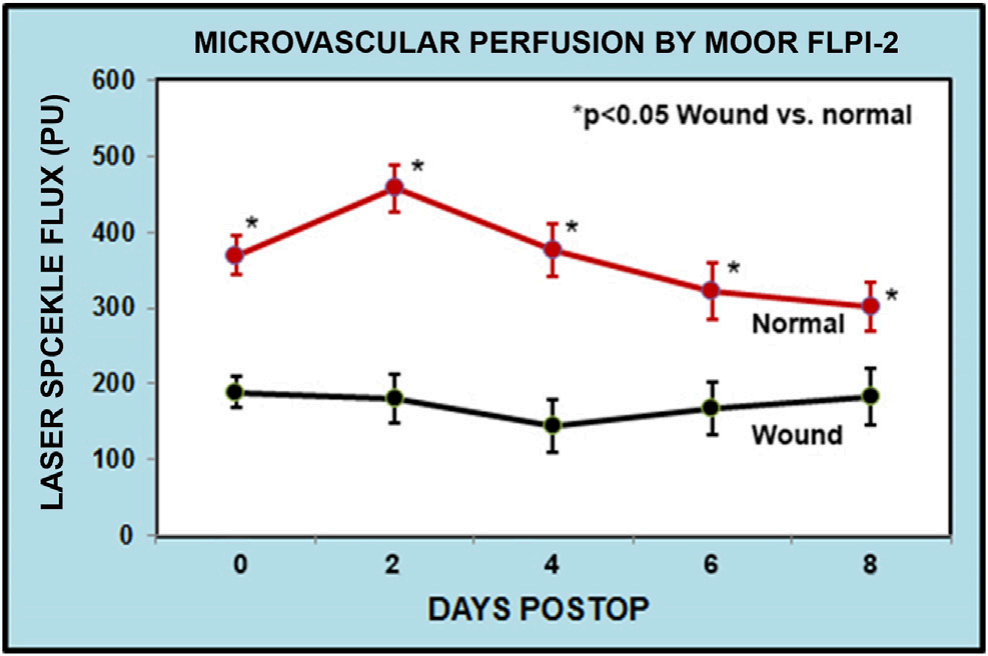
Graphical representation of flux perfusion in rabbit back skin incisional wounds. Measured skin blood flow between the incisional wounds and normal skin using Moor FLPI -2 perfusion imager. The ischemia difference was more significant at the early days which gradually decreased after 6–8 days. The FLPI-2 measurement is recorded as flux reading. The data were obtained from 8 to 10 different ROI (region of interest) sites as shown in Figure 1.

A reduced microvascular perfusion around the wound area was clearly seen by laser speckle contrast imager (Moor FLPI-2) and this was especially significant in the first week (Figure 1) Ischemia decreased gradually after a week but still existed in some wounds (Figure 2).

### Wound Tissue Tensile and Breaking Strengths

**FIGURE 3:**
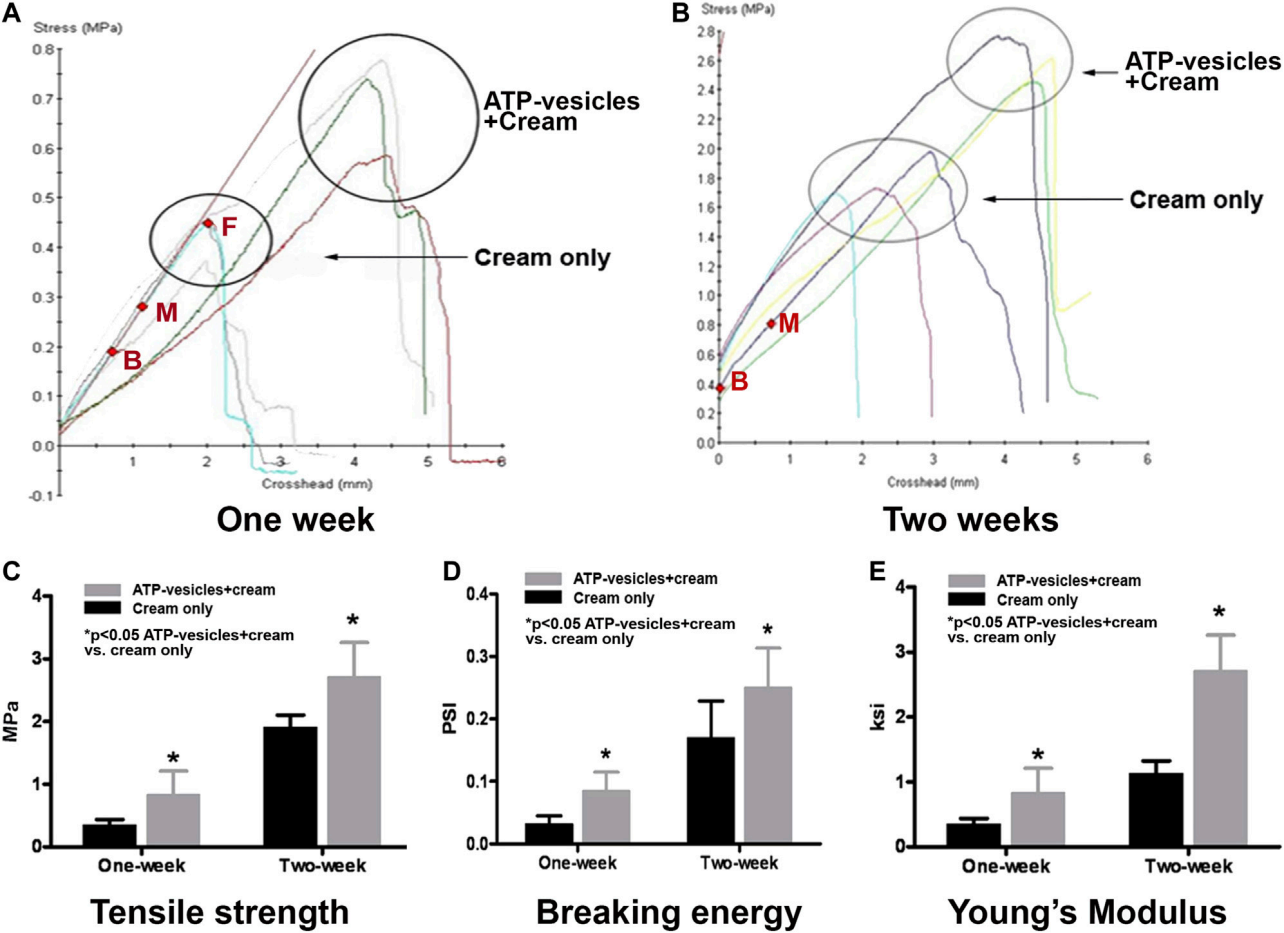
Biomechanical testing of wound tissues. **(A)**. At postoperative day 7. Tissue tensile strength has a linear relationship between load and elongation of the tissue up to the point of peak stress (from point B to point M). After that point, the relationship is no longer linear but breakage does not occur until further stretching (point F). **(B)**. At postoperative day 14, wounds have higher tensile strength than at day 7. **(C)**. Statistical comparison of wound tissue tensile strength between the experimental and control groups at postoperative days 7 and 14. **(D)**. Comparison of wound breaking energy at postoperative days 7 and 14. At postoperative day 7, the breaking energy in the experimental wounds was 198% of the value for the control wounds. At postoperative day 14, the breaking energy in the experimental group was 147% of the value for control wounds. **(E)**. Comparison of Young’s modulus (stress/strain, within elastic limit; i.e., Hooke’s law) of wound tissues between the two groups; the average value at postoperative day 7 in the experimental wounds was 242% of the value for the control wounds. At postoperative day 14, the average value in the experimental wounds was 249% of the value for the control wounds (Note: 1 psi = 6.895 MPa = 0.703 g/mm^2^).

At the seventh postoperative day, tensile strength was higher in the experimental group (treated with ATP-vesicles, 0.71 ± 0.16 MPa) than in the control group (treated with cream only, 0.35 ± 0.03 MPa, *p* < 0.05, Figure 3A). At 14 days, tensile strength was further increased and it was again higher in the experimental group (2.59 ± 0.30 MPa) than in the control group (1.68 ± 0.19 MPa, *p* < 0.05, Figure 3B). The difference is shown in Figure 3C.

Further stretching of the specimen eventually creates a force that the narrowed band cannot withstand, and the wound strip ruptures apart (Figure 3A point F). The force required to break the specimen is referred as its breaking energy. Significant differences were observed between the experimental and control groups at 7 days (0.085 ± 0.029 psi vs. 0.032 ± 0.013 psi, *p* < 0.05) and 14 days (0.251 ± 0.062 psi vs. 0.171 ± 0.058 psi, *p* < 0.05); these differences were similar in magnitude and pattern to the differences observed for tensile strength (Figure 3D).

However, these values (peak tensile strength and breaking energy) at both 7 and 14 days were still lower than that of normal skin (about 1/3 to 1/5 of normal skin at day 14). This is expected since a complete healing can take up to two years (Richardson, 2004).

### Wound Tissue Elasticity

Young’s modulus (also known as the Modulus of elasticity) is the numerical evaluation of Hooke’s Law; the ratio of stress to strain. Significant differences were observed between the experimental and control groups at postoperative day 7 (0.838 ± 0.132 ksi vs. 0.346 ± 0.033 ksi, *p* < 0.05) and 14 (2.719 ± 0.192 ksi vs. 1.092 ± 0.068 ksi, *p* < 0.05, Figure 3E).

### Microscopy Study

**FIGURE 4:**
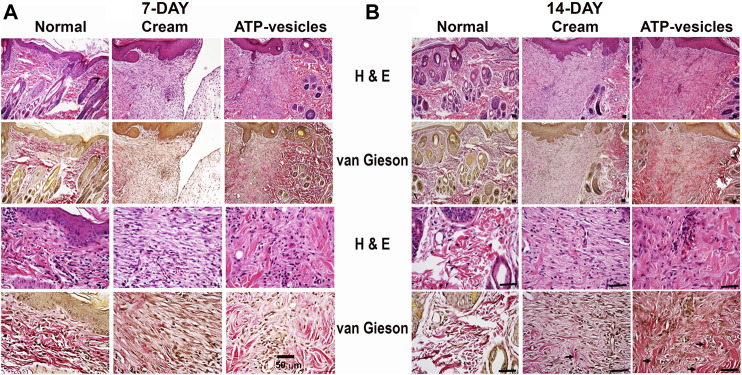
Hematoxylin/eosin and Van Gieson staining of wound tissues on postoperative day 7**(A)** and 14 **(B)**. The representative images show the wounds treated with ATP-vesicles have more intense collagen fibers (red color) than the wounds treated with neutral cream. The collagen fibers also exhibit a more organized pattern in ATP-vesicle-treated wounds on postoperative day 14. The arrows indicate positive staining of the collagen fibers. Scale bar is 50 μm.

Representative histological photomicrographs with H&E and van Gieson staining are shown in Figures 4A,B. A notable increase in collagen deposition along the healing line was observed on postoperative days 7 and 14 in the ATP-vesicle-treated wounds when compared with the control group, indicating better healing in response to ATP-vesicles. The collagen fibrils in ATP treated wounds were denser when compared with control.

### Macrophage Accumulation

**FIGURE 5:**
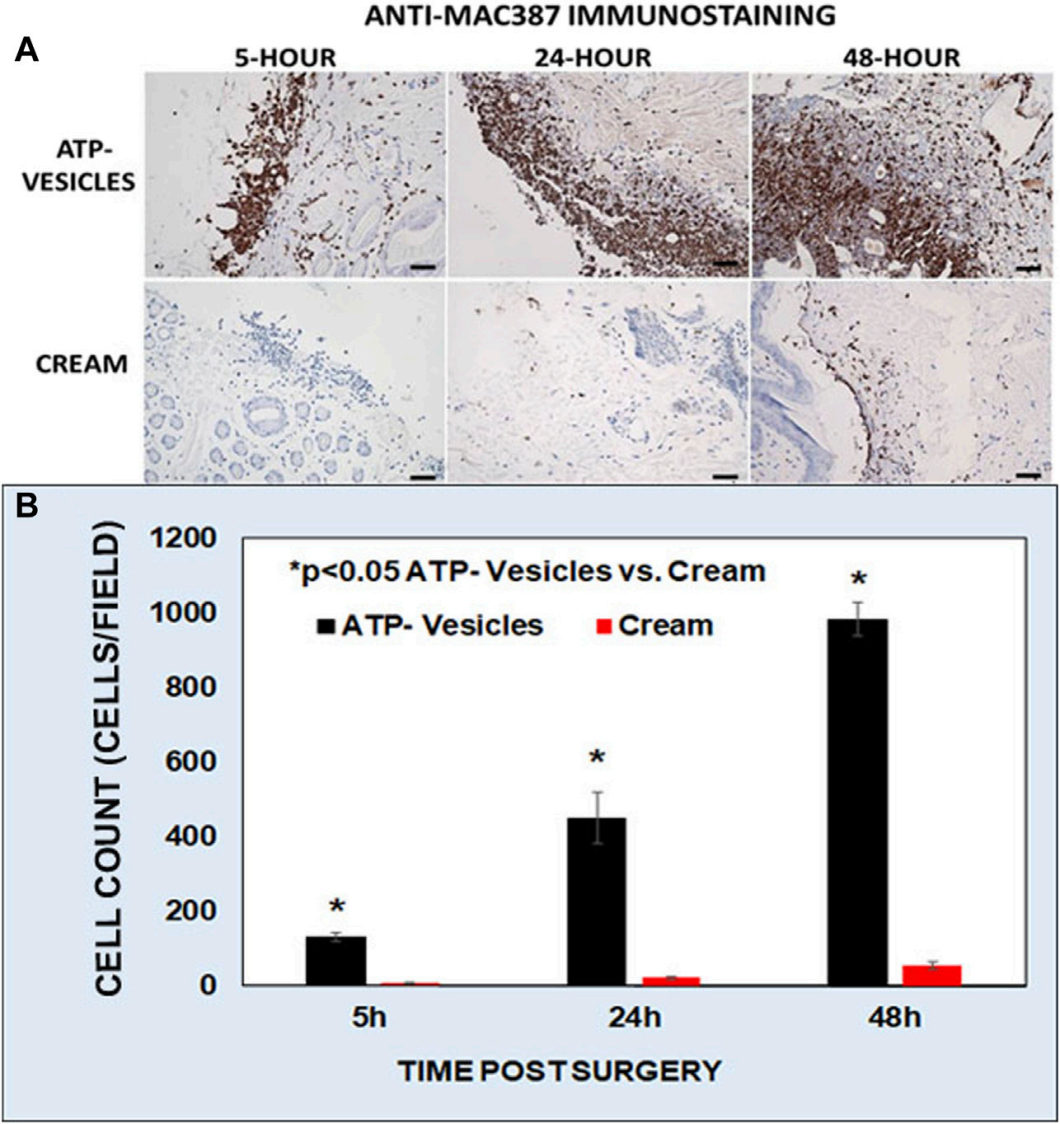
A representative immunohistochemistry comparison of macrophage accumulation between the two groups at 5, 24, and 48 h after surgery. **(A)**. Brown color indicates anti-MAC387 positive immunostaining of macrophages. In this study ATP-vesicle-treated wounds showed significant macrophage accumulation whereas the control wounds have no such accumulation. Scale bar is 50 μm. **(B)**. Graphical representation of the anti-MAC387 positive cells between the two groups.

One very special finding was, in the wounds treated by ATP-vesicles, very early and massive macrophage accumulation occurred on the wound edge as shown by macrophage antisera (Anti-Mac387). The accumulation started as early as 5 h after surgery, and the accumulation continued thereafter (Figure 5A). The control wounds showed no such accumulation. Wound cell calculation indicated that the wounds treated by ATP-vesicles, macrophage number was 6–8 times higher at days 1 and 2 than that in the control wounds (Figure 5B). However, this number decreased quickly after 3 days and became lower than the control wounds at days 6. The representative images of very early macrophage accumulation are shown by anti-Mac387 immunostaining (Figure 5A).

### Collagen Staining

**FIGURE 6:**
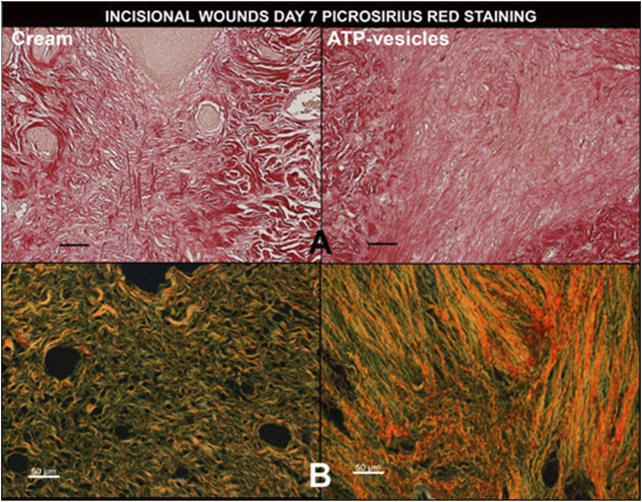
A representative comparison of wound collagen by picrosirius red staining 7 days after surgery between the two groups. **(A)**. Under a regular light microscope, ATP- vesicle-treated wounds show some large sized and regimented collagen fibrils (bright red), whereas the control wound has less and poorly-organized collagen. **(B)**. Under circular polarized microscope, the study wound shows higher type I collagen contents (stained yellow and orange) while the control wound has fewer and mostly type III collagen (stained green). Scale bar is 50 μm.

Wound collagen staining by picrosirius red indicated massive and well-organized collagen accumulation in wounds treated with ATP-vesicles, while the control wounds showed much less accumulation (Figure 6A). Further analysis by circular polarizing microscopy indicated more and better-organized type I collagen (yellow to orange color) and some type III collagen (green color) in the ATP-vesicle-treated wounds than in the cream alone treated wounds (Figure 6B).

**FIGURE 7:**
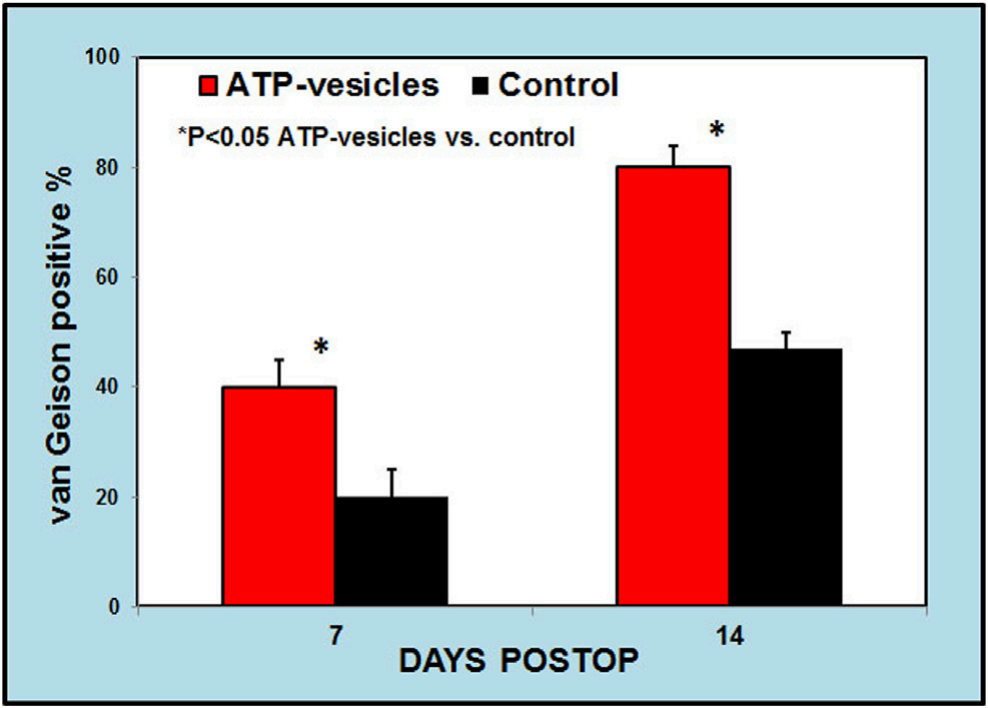
A. Morphometric analysis of collagen to total wound bed ratio by van Gieson staining. This analysis indicates higher collagen contents in ATP-vesicle treated wounds than in cream-treated controls at day 7 and day 14. (Mean ± SD of six independent experiments *p* < 0.05).

Morphometric analysis of Van Gieson-stained wound tissues indicated that the experimental group had 50.2 and 80.5% Van Gieson-positive staining on days 7 and day 14, respectively, compared to 19.8 and 50.2%, respectively, for the control group (Figure 7).

### Collagen Generation by Macrophages

**FIGURE 8:**
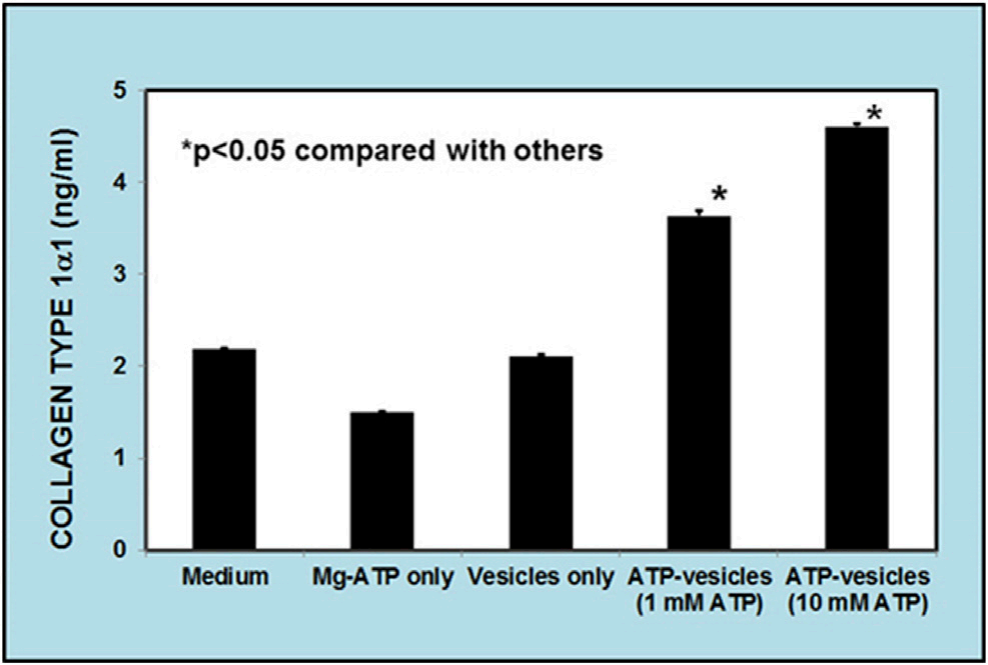
Enzyme-linked immunosorbent assays (ELISA) of soluble collagen secreted from human macrophages in response to different treatments. Macrophages produce collagen, but addition of ATP-vesicles to the macrophages increased collagen production by two-fold while free Mg-ATP and lipid vesicles alone did not show any enhanced effect. (Mean ±SD of three independent experiments *p* < 0.05).

**FIGURE 9:**
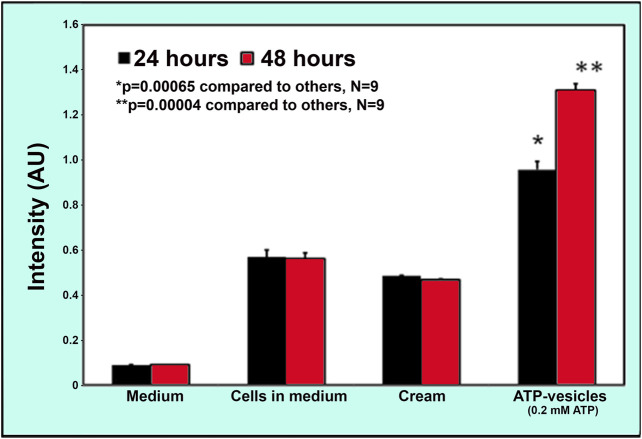
Comparison of collagen production by macrophages *in vitro* when treated with ATP-vesicles and the control. Addition of 0.2 mM ATP-vesicles to the macrophages in culture for 24 and 48 h increased the collagen release when compared with the control cream alone treatment (Mean ±SD of nine independent experiments *p* < 0.001).

Although the possibility of direct macrophage collagen production *via* alternative activation was proposed more than 2 decades ago (Vaage and Harlos, 1991; Anghelina et al., 2006; Wynn, 2008) it is still largely dismissed by most scientists, especially in wound healing (Vaage and Lindblad, 1990; Schnoor et al., 2008). To further confirm this possibility, we performed an isolated macrophage culture to exclude the role of other cells. Soluble collagen production was measured at 24 h in human macrophage cultures. We first compared the capability of collagen production by ATP-vesicles and their distinct components—free lipid vesicles and free Mg-ATP. Results indicated that the addition of ATP-vesicles (at an ATP concentration of 1–10 mM) increased collagen production while neither free Mg-ATP nor lipid vesicles alone produced any more collagen than the culture medium (Figure 8). We then compared ATP-vesicles with the control for their ability to enhanced production. Results indicated that the neutral cream did not promote collagen production while the addition of ATP-vesicles doubled the collagen production (Figure 9).

## Discussion

The major findings in the study include the following: 1) Application of ATP-vesicles enhanced incisional wound tissue strengths as indicated by increased tensile strength, breaking energy, and elasticity. These are the major factors in incisional wound healing; 2) the increased healing strength was accompanied by very early and massive macrophage accumulation and *in situ* proliferation; 3) The use of ATP-vesicles also caused macrophage M2 polarization and direct collagen production which is a key player in the enhanced incisional wound healing strength. These findings have not been reported previously in incisional wound healing.

Surgeons have taken great precautions to reduce the occurrence of wound failure because of the presumed individual surgeon’s culpability and because of the gloomy outcome of these complications by treating the above co-morbidities aggressively before and after surgery (Pickleman et al., 1999). Studies on selection of suture material, knotting techniques, depth of bite, number of sutures, and site of incision have all obtained some promising results (Moossa et al., 1997). Despite thousands of dressings developed in the past century for wound care, none has shown an ability to improve surgical wound tensile strength, including the only FDA-approved prescription growth factor, Regranex (Dinah and Adhikari, 2006; Barrientos et al., 2008; Barrientos et al., 2014). In 1939, Dr. Norris wrote his comment on wound dehiscence: “The sad commentary on its present status is the fact that the literature continues to report its incidence relatively unchanged” (Norris, 1939). The even sadder fact is, after 80 years, this statement is as true today as it was. In fact, the incidence of wound breakdown is increasing with the intense growth of bariatric surgery (White et al., 1977; Franz et al., 2007). This discouraging outcome has in turn discouraged new development, and research work on minimizing wound breakdown is now sporadic at best (Klinge et al., 2006). Tissue failure occurs in a wound in the biochemically active zone nearby the acute wound edge where the sutures are located. Proteases activated during normal tissue repair cause a loss of native tissue integrity, especially in the early postoperative days (Franz et al., 2007). The main process involved in incisional wound healing is the re-creation of mechanical strength by restoration of tissue continuity. Surgical incisions are acute wounds and they activate the healing process in a similar manner to excisional wounds, but they are physiologically different from excisional wounds with respect to the healing process. Excisional wound closure requires granulation tissue formation intervened through angiogenesis and epithelialisation. In contrast, the surgical incisional wound healing depends more on the qualitative and quantitative formation of the extracellular matrix, especially tissue collagen production and cross-linking, rather than angiogenesis and reepithelialization (Posma et al., 2007).

Providing energy to acute wound tissue is one approach for reducing breakdown. This is because the wound space becomes hypoxic, hypoglycemic, and acidotic (Adzick, 1997), and even the most uncomplicated wounds contain focal areas in which pO_2_ approaches zero (Allen et al., 1997). A combination of the following factors contributes to this process: 1) hemostasis curtails blood flow directly to the site of injury, as the creation of fibrin clots in the local vessels stops bleeding (Levenson and Demetriou, 1992); 2) a hypo perfusion state occurs often in the postoperative period (Jonsson et al., 1987); 3) sutures placed to close the wound may further compromise blood supply (Cherry et al., 2000); and 4) the accumulation of inflammatory cells in the wound tissue significantly increases metabolic energy consumption (Adzick, 1997; Lin et al., 1998). Wound hypoxia also increases the risk of infection because it impairs the function of the neutrophils, lymphocytes, and macrophages needed for wound repair and bactericidal functions (Gottrup 2004).

One primary result of tissue hypoxia/ischemia is a significant reduction in cellular energy reserves (Im and Hoopes, 1970), which is essential for every phase of the wound healing (Wang et al., 1990). Synthesis of proteins, such as collagen, which occurs by linking of individual component amino acids with peptide bonds, requires a particularly large expenditure of chemical energy (Stadelmann et al., 1998). Furthermore, inflammatory cells also cannot function without adequate energy supply. This is probably one reason that Regranex was shown effective in many animal studies but has not shown consistent results in wound healing because it cannot penetrate the wound to reach the cells (Barrientos et al., 2014). The ATP-vesicles used in this study were previously tested in various cell culture and tissue preservation experiments, and an equally efficient delivery of Mg-ATP into the cytosol of wound cells therefore likely occurred in the wound area in the present study (Chien 2010a; Sarojini et al., 2017).

Our excisional wound study revealed a unique phenomenon when ATP-vesicles were used: very early and massive macrophage accumulation, *in situ* proliferation, and M2 polarization (Wang et al., 2009a; Wang et al., 2010; Howard et al., 2014; Sarojini et al., 2017; Mo et al., 2020). The M2 polarized macrophages produce collagen directly to enhance healing at early time. The provided energy also maintains their survival and function of the wound cells during the early ischemic stage (Sarojini et al., 2017; Mo et al., 2020). The healing events occurred in the current study also shows the same progression.

Tissue tensile strength of surgical wounds has characteristics that resemble an exponential curve (Muehlberger et al., 2005). In the early days of the wound repair process, moderate increase is seen in the tensile strength. An acceleration of the healing process, accompanied by a rapid increase in the tensile strength, reaches a steady state at about eight weeks and reaches a maximum peak after more than one-year post injury (Hahler, 2006). Our study duration was not this long, as most wound breakdown (the focus of the present study) occurs in the first week after surgery (Cliby, 2002). Nevertheless, treatment with ATP-vesicles significantly enhanced tensile strength, providing new hope for enhancing the healing strength of incisional wounds. Similar results were seen for breaking strength and Young’s modulus measurements, indicating an increase in wound tissue elasticity. These are the most important characteristics for incisional wounds to withstand bursting pressure, which is the key to wound dehiscence.

The findings in the current study indicated a direct link between energy supply and wound strength, which was accompanied by significant macrophage accumulation and collagen production. The phenomenon seen here has not been reported in the past. Because wound healing is an extremely complicate process with numerous factors involved, the mechanism behind this improvement is still not totally unclear. To achieve the results seen in our study, at least three pathways need to be involved: 1) massive stem/progenitor cell trafficking and leukocyte chemotaxis caused by purinergic receptor activation (Junger, 2011); 2) improved cell survival and function due to intracellularly delivered energy because energy is specially required for macrophages to exert their bactericidal function as well as their tissue remodeling effects; and 3) enhanced collagen production and cross-linking by activated macrophages resulting in reduced tissue separation and infection. At the same time, activated macrophages secret many factors such as MCP-1 (Kim et al., 2012), which induce further cell accumulation; this was not seen in control wounds. Our excisional wound healing has shown this effect (Sarojini et al., 2017). This type of a reciprocal feedback cycle is probably the main reason for the extremely rapid macrophage accumulation. The intracellularly delivered ATP is needed to keep these newly arrived cells alive and functioning (Figure 9).

**FIGURE 10:**
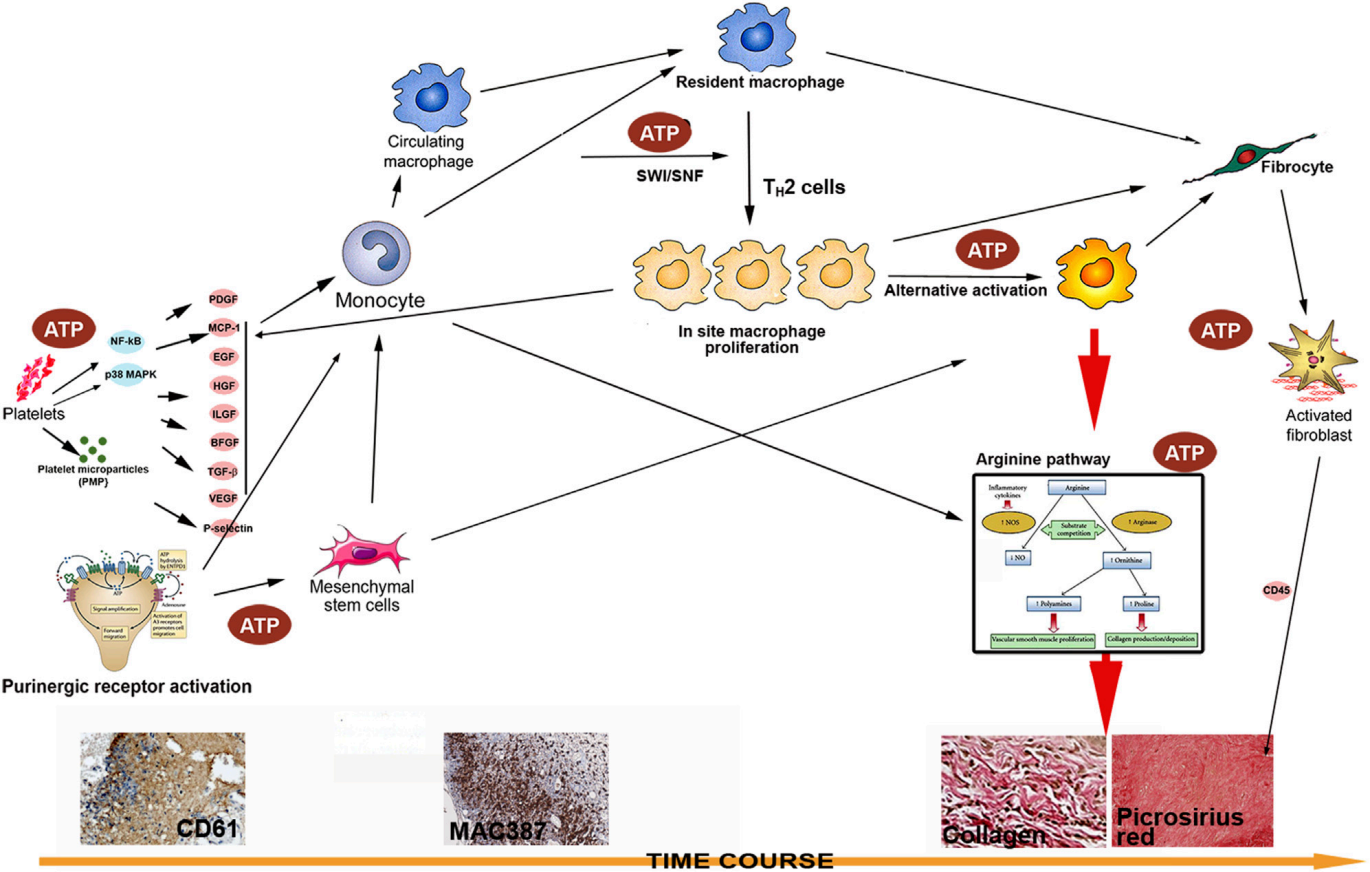
A schematic illustration of possible mechanisms through which intracellular ATP delivery augments incisional wound healing: 1) ATP promotes stem/progenitor cell trafficking, leukocyte chemotaxis, and platelet activation *via* both purinergic receptor activation and energy enhancement, resulting in early platelet trafficking and massive macrophage accumulation; 2) Activated macrophages produce many factors to further encourage trafficking, *in situ* proliferation, and M2 polarization; 3) Activated macrophages produce collagen directly *via* alternative activation, leading to enhanced and rapid healing.

Macrophages are essential components of innate immunity and they play a central role in inflammation and host defense (classic activation). However, these cells also fulfill homeostatic functions beyond defense and play a major role in tissue remodeling (alternative activation) (Sica and Mantovani, 2012). In other diseases, such as tumor encapsulation, solid organ fibrosis, muscular dystrophy, and atherosclerosis the capability of macrophages to produce collagen is well proven. In contrast, their role in wound healing is not well studied (Diegelmann and Evans, 2004). The likelihood of macrophages producing collagen directly was proposed 2 decades ago, but this idea is still not accepted by some scientists (Vaage and Lindblad, 1990; Schnoor et al., 2008). Collagen production by alternative activation of macrophage is believed to occur *via* the arginine pathway that leads to proline production—a major component of collagen (Classen et al., 2009). In the present study, wound treated by ATP-vesicles showed not only higher collagen contents but they are also much better organized. To further confirm this effect, cultured macrophages produced collagen by themselves within 24 h. However, addition of ATP-vesicles enhanced collagen production, while free Mg-ATP, free lipid vesicles, and cream alone had no such effect. At this early stage, most of the macrophages still maintained their classic phenotype and had not begun to transform into fibroblasts. This result provides new evidence for direct collagen production by macrophages. At this time, our understanding of macrophage plasticity is still very limited—a full spectrum of the roles played by this highly plastic cell in the wound healing process will gradually emerge as more studies are performed. The proposed cascade is shown schematically in Figure 10. Further extensive exploration is required to fully delineate all the pathways involved in this process, but our study has provided initial evidence to support this cascade.

Whether the results obtained in this study can be extrapolated to full surgical abdominal wound healing requires further study. In normal abdominal incisions, the musculoaponeurotic layer and the peritoneum play major roles in securing the incisional wounds and preventing evisceration while the skin incision holds intact (Moossa et al., 1997). To reduce the abdominal herniation, a drug has to penetrate deep into the peritoneal layer. Our previous skin penetration studies already indicated that Mg-ATP encapsulated in lipid vesicles had an increased penetration through the skin (Chien, 2010a). Our excisional wound study confirmed that the ATP-vesicles penetrated through the rabbit ear cartilage, causing massive macrophage accumulation underneath the ear cartilage (Howard et al., 2014). Of particular interest, ATP-vesicles-treated wounds healed faster than wounds treated with other dressings (Wang et al., 2009a; Wang et al., 2010), including the only FDA-approved prescription growth factor for wound care—Regranex (Howard et al., 2014). A possible role of macrophages in this process was also explored (Sarojini et al., 2017), and the results are in agreement with the current experiments.

To our knowledge, this study is the first report to use intracellular energy delivery to enhance incisional wound healing, and one of very few aimed at prevention rather than treatment of surgical wound dehiscence. Rabbits can regenerate ear cartilage when the perichondrium is intact, but their skin regeneration capability is similar to that of other mammals (Williams-Boyce and Daniel, 1986). Due to the unresolved problem of surgical wound breakdown and the scarcity of similar studies, our results have provided some encouragement that although prevention of wound dehiscence is difficult, it may not be totally impossible.

## Data Availability

The original contributions presented in the study are included in the article/Supplementary Material, further inquiries can be directed to the corresponding author.
