# Supplementary material for: Enhanced Skin Incisional Wound Healing With Intracellular ATP Delivery via Macrophage Proliferation and Direct Collagen Production
Source: Front Pharmacol. 2021 Jun 16;12:594586. doi: 10.3389/fphar.2021.594586 (PMC8241909; doi:10.3389/fphar.2021.594586)
Supplement: Supplementary file 1 [file Image2.pdf]

Figure S2. Skin microvascular perfusion measurements

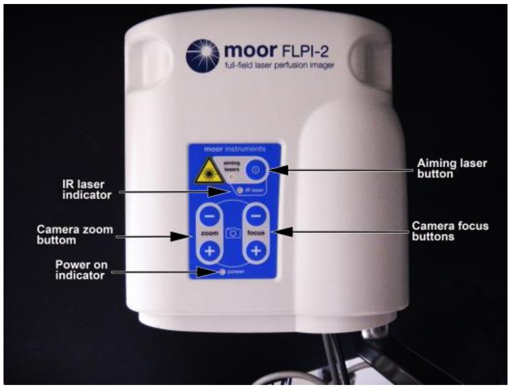

Figure. S2a. The front view of Moor FLPI-2. All the buttons are in front for easy to use and control.

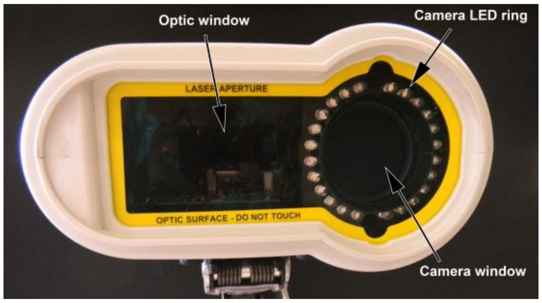

Figure. S2b. The bottom view of Moor-FLPI-2 showing the camera and optic windows.

In the past decades, multiple instruments for blood perfusion have been developed based on laser Doppler or laser speckle contrast techniques. However, the early instruments were highly unreliable to give the correct results. For example, a small change in the room light can significantly alter the image resulting in incorrect results. For this reason, different laser speckle systems wrote their own software to calculate the contrast value hoping to reduce the variation. However, this did not change the final results because all formulations are based on the preliminary measured date. The Moor FLPI-2 was developed in recent years which has incorporated multiple new techniques. It is more stable now and it has become a standard for tissue perfusion measurements We tested it many times in our study and even under different light conditions the results are very similar. We always test our wound skin perfusion with various equipment in order to obtain the most accurate oxygenation. The equipment we have used includes TiVi-8000 (Wheels Bridge’s, Linköping, Sweden), FLIR-C3 thermal camera (Teledyne FLIR, Thousand Oaks, CA), OxyLite Pro oxygen tension monitor (Oxford Optronix, England), Radiometer Tina TCM4 (Radiometer, Copenhagen), and the Moor FLPI-2 Laser Speckle Contrast Imager (Moor Instruments, England). Each one has its own specific function suitable for different purposes. However, Moor FLPI-2 has the advantages of easy to use, totally noninvasive, easy to focus, faster to obtain results, and more reliable (Figure. S2a and S2b).

Principle of operation

10.2 Principle of Operation

The moorFLPI-2 measures tissue perfusion by performing contrast analysis on images acquired from a CCD video camera. The analysis can be performed using either low resolution spatial processing or high resolution temporal processing. Spatial processing involves the analysis of the intensity variation within small groups of pixels (typically 5x5 pixels) within a single frame of video data. Temporal processing involves the analysis of the intensity variation of single pixels over a number of frames (typically at least 25) of video data. In general, temporal processing is capable of producing images with high resolution at relatively low speed, whilst spatial processing produces images with reduced resolution at high speed (up to 25 frames per second).

The same image processing algorithm is used for both approaches to the analysis. For each pixel in the flux image the speckle contrast ***K***, of a number of pixels in the video image is calculated. For spatial processing this calculation is performed on a square group (or kernel) of 5x5 pixels in a single frame of image data and for temporal processing the calculation is performed on a single pixel location over a number of frames of image data (referred to as a temporal filter). Speckle contrast K is defined as the ratio of the standard deviation σ to the mean </> of pixel intensity values within each group:

$$K = \frac{\sigma}{\langle I \rangle}$$

Assuming Brownian motion with Lorentzian power spectrum of the velocity distribution the relationship between speckle contrast ***K***, correlation time T_c, and camera integration time ***T*** can be expressed as:

$$(1) \qquad K = \frac{\sigma}{\langle I \rangle} = \left\{ \frac{\tau_c}{2T} \left[1 - e^{\left(\frac{-2T}{\tau_c}\right)} \right] \right\}^{1/2}$$

The speckle contrast ***K*** should vary between 0 (no speckle, very high perfusion) and 1 (fully developed speckle, very low perfusion). In practice, measured speckle contrast never exceeds a value of 0.5, even when a stationary target is imaged and a fully developed speckle pattern is never observed, irrespective of integration time. Over this reduced range of speckle contrast values (***K*** = 0 to 0.5) the $1 - e^{\left(\frac{-2T}{\tau_c}\right)}$ term in equation 1 is very close to unity allowing simplification of equation 1 to:

$$(2) \qquad K = \frac{\sigma}{\langle I \rangle} = \left(\frac{\tau_c}{2T} \right)^{\frac{1}{2}}$$

The correlation time $T_c = 1/(a k_o v)$ were ***a*** is an unknown factor related to the Lorentzian width of the scattered spectrum and the scattering properties of the tissue, ***v*** is the mean velocity and k_o is the input light wave number. If we assume that perfusion is proportional to the mean velocity ***v*** then perfusion is inversely proportional to the correlation time. Applying this assumption to equation (2) gives blood flow in terms of speckle contrast (for fixed exposure time):

$$(3) \qquad Flux \propto \left(\frac{\langle I \rangle}{\sigma} \right)^2$$

The moorFLPI-2 is standardised around an integration time of 20ms. As speckle contrast is dependant on integration time, shorter integration times will give lower measured perfusion values but the potential to measure higher maximum perfusion whilst longer integration times will tend to give higher perfusion values (effectively providing increased sensitivity) but reduced measurement range. This is similar to bandwidth selection in the frequency domain for laser Doppler perfusion assessment.

The above document shows that the final perfusion units Flux is already the result of calculation, not the raw data.

Specifications

The specifications of Moor FLPI-2 are shown in the following table.

System Specifications

Measured parameters	**Flux** (tissue perfusion) Range: 0-5000 PU Resolution: 1 PU Accuracy: ±10%<br>**DC** (intensity) Range: 0-255 AU Resolution: 1 AU Accuracy: ±1 AU<br><br>Sampling rate (all parameters): 25Hz
Image resolution	150 x116 pixels (Spatial processing)<br>752 x 580 pixels (Temporal processing)
Image area (depends on distance and zoom)	(5.6 x 7.5mm) to (15 x 20cm)
Working distance	10 - 38cm, between scan head and measurement site
Image area measurement accuracy	±0.5mm or ±5%, whichever is greater
Distance measurement accuracy	±5%
Type of protection against electric shock	Class I
Degree of protection against electric shock	Non-patient contact, no applied part
Medical Devices Directive classification	Class IIa
Power source	AC mains, Universal switched mode, 100-230V, 50-60Hz
Power consumption	30VA
Degree of protection against flammable anaesthetics	Not Protected
Degree of protection against ingress of liquid	Not protected, IPX0
Mode of operation	Continuous
Operating temperature	15 – 30ºC
Storage and transportation conditions	Temperature: 5 – 50ºC<br>Humidity: 20 – 80% RH (excluding condensation)
Scan head dimensions	(WxHxD cm) 23 x 12 x 25 Weight 2.3kg
Power supply dimensions	(WxHxD cm) 10 x 5.3 x 16 Weight 0.7kg
Methods of mounting	VESA compliant monitor arm (75mm square fixing centres)<br>Photographic tripod or enlarger stand (1/4" UNC fitting)
Laser classification	Class 1 Laser Product, IEC 60825-1:2007
Laser output	*Measurement Laser*<br>Wavelength: 785nm ± 10nm<br>Maximum power: 50mW, Accessible power below IEC 60825-1:2007 Class 1 limits<br><br>*Aiming Lasers*<br>Wavelength: 650nm<br>Maximum power: 0.12mW per laser
Standards of conformance	EN60601-1:2005<br>EN60601-1-2:2007<br>IEC 60825-1:2007<br>IEC 62471:2006<br><br>Complies with CFR 1040.10 and 1040.11 except for deviations pursuant to Laser Notice No. 50 dated June 24, 2007
Minimum PC specification	Dual core Pentium™ 4 processor, 2GHz or higher compatible CPU.<br>2GB RAM<br>Super VGA monitor (1024 x 768 resolution or higher)<br>100GB of available hard drive space<br>Windows™ XP or higher<br>1 USB port and 1 FireWire (IEEE 1394) port

*Moor Instruments reserves the right to change specification without notice

The following Settings are used in our measurements

In this study, the following settings were used:

Measurement parameters: Flux (tissue perfusion): range (0-5000 PU), resolution (1 PU), accuracy (±10%).

Processing model: High resolution/low speed.

Imaging display frequency: 25 Hz.

Image resolution: 752x580 pixels (temporal processing).

Laser output: Aiming laser wavelength (650 nm), measurement laser wavelength (785 nm),

Duration of scanning: 100 images.

Limitations of Moor FLPI-2

The main limitation is its very superficial measurement. The microvascular in the skin is only measured at the 1-3 mm while using spatial or temporal process, which cannot detect the whole incisional wounds in larger animals or humans. Another limitation in animals is their very rapid hair growth. Figure. S2c shows the rabbit back wounds. At day 4, the hair grows back to form a thick layer. The newly formed hair can significantly affect the imaging results.

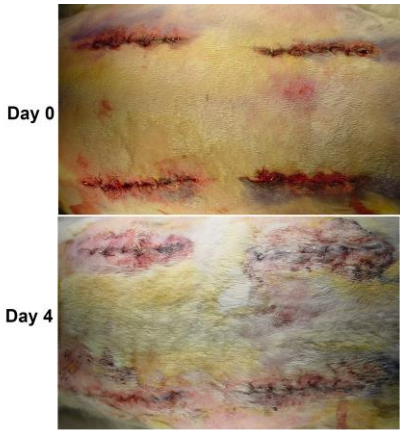

Figure. S2c. An example of back wounds in a rabbit. The hair was shaved clean during surgery. At day 4, it grows back to a thick layer, which makes skin perfusion imaging unreliable.
