# Supplementary material for: Enhanced Skin Incisional Wound Healing With Intracellular ATP Delivery via Macrophage Proliferation and Direct Collagen Production
Source: Front Pharmacol. 2021 Jun 16;12:594586. doi: 10.3389/fphar.2021.594586 (PMC8241909; doi:10.3389/fphar.2021.594586)
Supplement: Supplementary file 3 [file DataSheet1.docx]

# Figure S1. Skin microvascular perfusion measurements


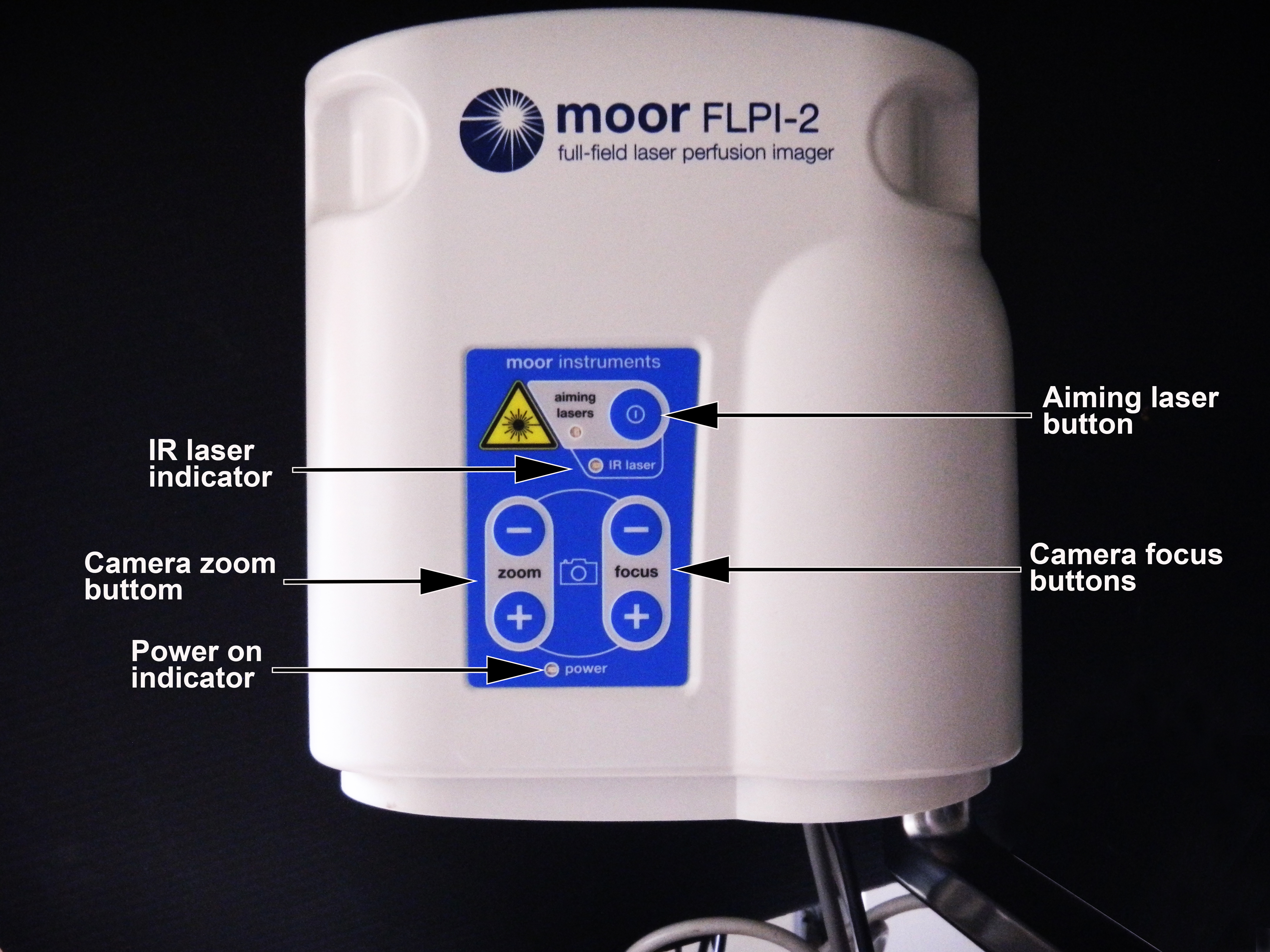


Figure. S1a. The front view of Moor FLPI-2. All the buttons are in front for easy to use and control.


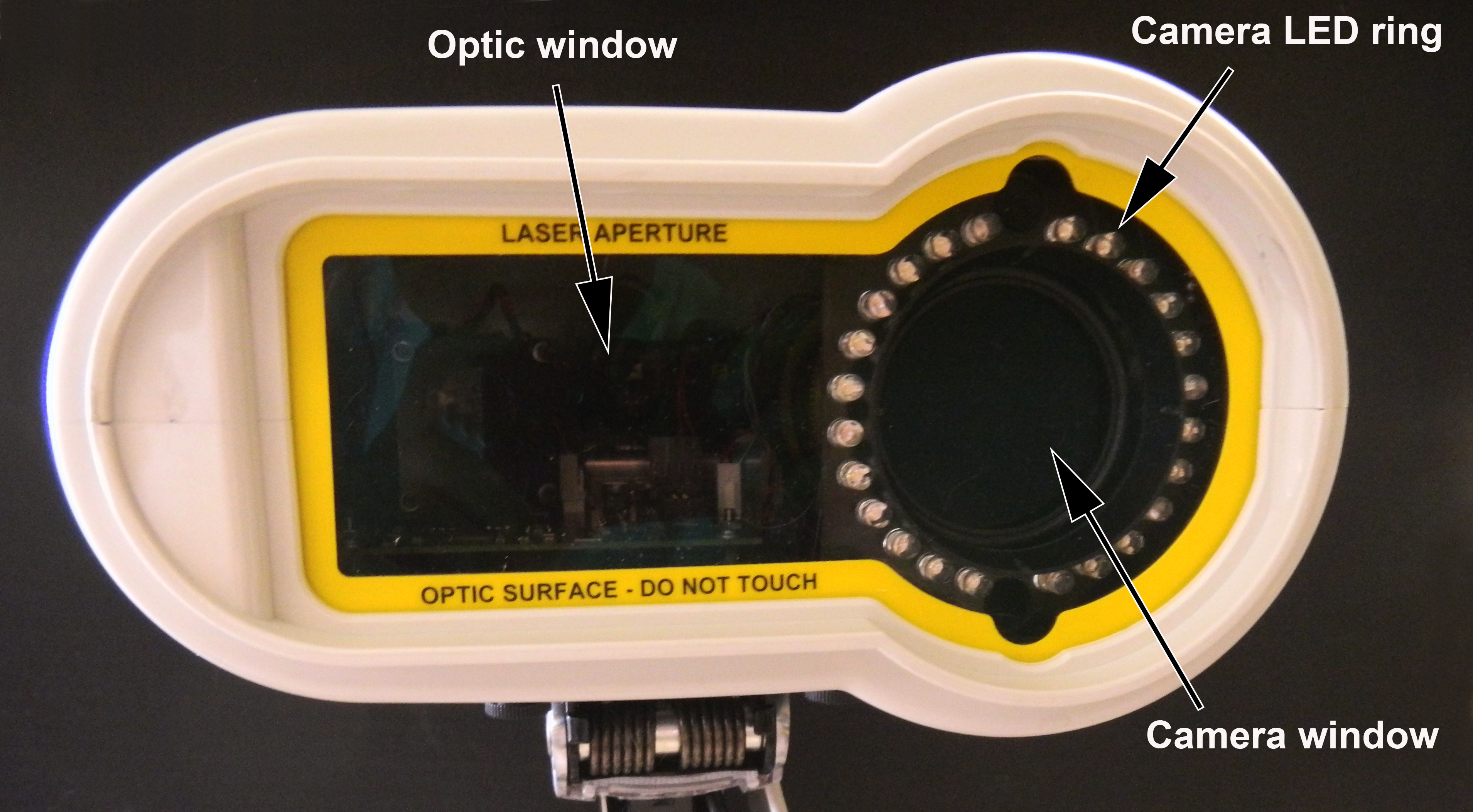


Figure. S1b. The bottom view of Moor-FLPI-2 showing the camera and optic windows.

In the past decades, multiple instruments for blood perfusion have been developed based on laser Doppler or laser speckle contrast techniques. However, the early instruments were highly unreliable to give the correct results. For example, a small change in the room light can significantly alter the image resulting in incorrect results. For this reason, different laser speckle systems wrote their own software to calculate the contrast value hoping to reduce the variation. However, this did not change the final results because all formulations are based on the preliminary measured date. The Moor FLPI-2 was developed in recent years which has incorporated multiple new techniques. It is more stable now and it has become a standard for tissue perfusion measurements We tested it many times in our study and even under different light conditions the results are very similar. We always test our wound skin perfusion with various equipment in order to obtain the most accurate oxygenation. The equipment we have used includes TiVi-8000 (Wheels Bridge’s, Linköping, Sweden), FLIR-C3 thermal camera (Teledyne FLIR, Thousand Oaks, CA), OxyLite Pro oxygen tension monitor (Oxford Optronix, England), Radiometer Tina TCM4 (Radiometer, Copenhagen), and the Moor FLPI-2 Laser Speckle Contrast Imager (Moor Instruments, England). Each one has its own specific function suitable for different purposes. However, Moor FLPI-2 has the advantages of easy to use, totally noninvasive, easy to focus, faster to obtain results, and more reliable (Figure. S1a and S1b).

# Principle of operation


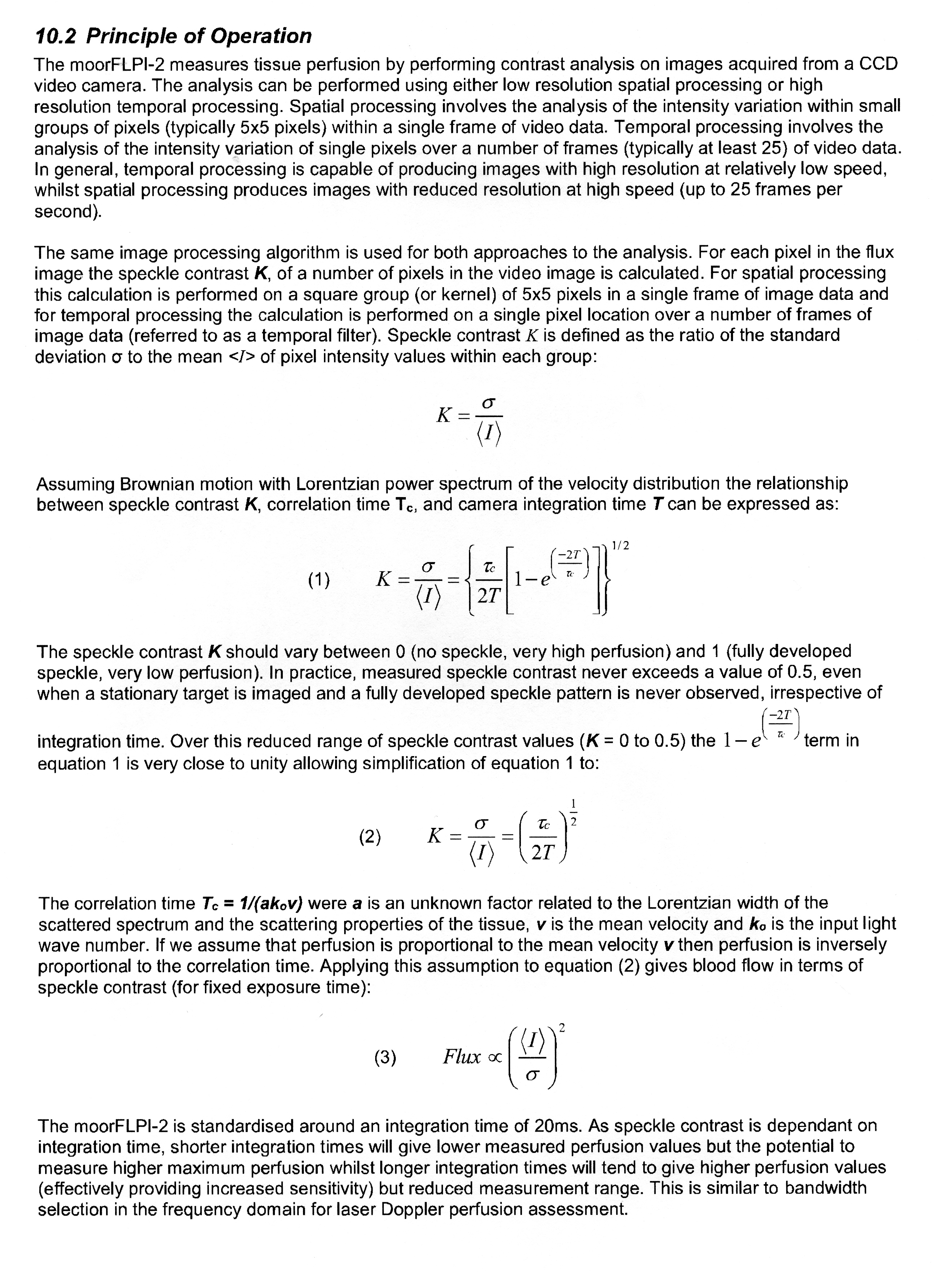


The above document shows that the final perfusion units Flux is already the result of calculation, not the raw data.

# Specifications

The specifications of Moor FLPI-2 are shown in the following table.
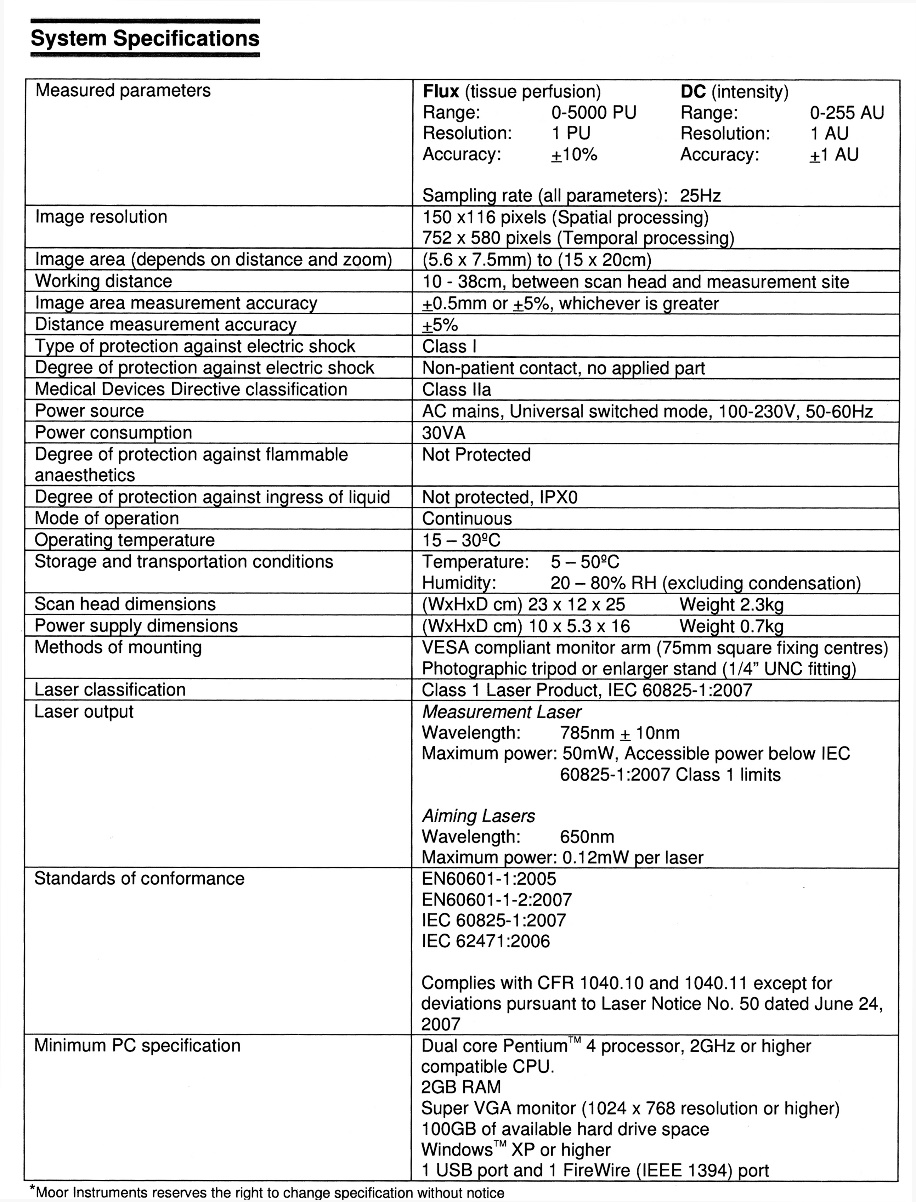


# The following Settings are used in our measurements

In this study, the following settings were used:

Measurement parameters: Flux (tissue perfusion): range (0-5000 PU), resolution (1 PU), accuracy (±10%).

Processing model: High resolution/low speed.

Imaging display frequency: 25 Hz.

Image resolution: 752x580 pixels (temporal processing).

Laser output: Aiming laser wavelength (650 nm), measurement laser wavelength (785 nm),

Duration of scanning: 100 images.

# Limitations of Moor FLPI-2


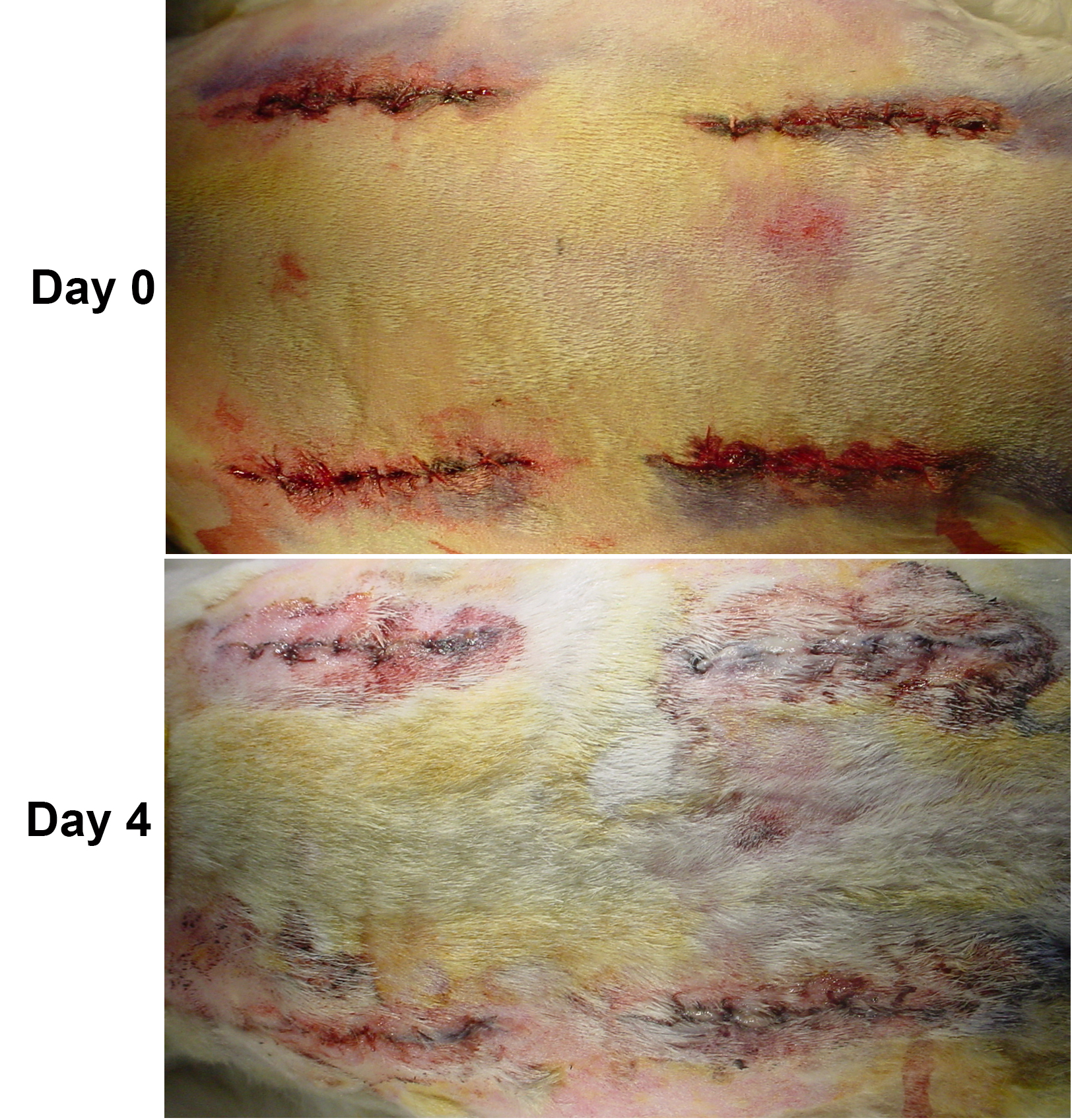


Figure. S1c. An example of back wounds in a rabbit. The hair was shaved clean during surgery. At day 4, it grows back to a thick layer, which makes skin perfusion imaging unreliable.

The main limitation is its very superficial measurement. The microvascular in the skin is only measured at the 1-3 mm while using spatial or temporal process, which cannot detect the whole incisional wounds in larger animals or humans. Another limitation in animals is their very rapid hair growth. Figure. S1c shows the rabbit back wounds. At day 4, the hair grows back to form a thick layer. The newly formed hair can significantly affect the imaging results.
