# Supplementary material for: Enhanced Skin Incisional Wound Healing With Intracellular ATP Delivery via Macrophage Proliferation and Direct Collagen Production
Source: Front Pharmacol. 2021 Jun 16;12:594586. doi: 10.3389/fphar.2021.594586 (PMC8241909; doi:10.3389/fphar.2021.594586)
Supplement: Supplementary file 4 [file Image1.pdf]

Figure. S1. Aluminum Skin Strip Mold

Our understanding of the complexity of skin wound healing has increased in recent years due to the use of various, highly reproducible animal models and testing equipment. Biomechanical assays serve as useful techniques to determine the extent of skin wound healing and provide insight into the functionality of repaired skin with different treatments. However, there are no standard tensile test protocols at this time. For this reason, investigators use different test specimen geometries and tensile stretching rates to measure tensile properties of skin wounds. The most common and simplest assay is the tensile test. However, with the improvement of instrument design software expansion, more parameters can be obtained which provide more detailed analyses of the healing quality of different wounds. It is critical to understand that the results may vary depending on the animal model, type of the skin, and the specimen geometry.

Testing the incisional wounds, it is critical to have the skin strips the same size, align the incision at the center of the strip, and the direction of the incision must be perpendicular (90°) to the strip cutting line. In many studies, the skin strips are very narrow (such as 2 mm). This narrow strip has a potential problem: In thick-skin animals, it is highly possible to result in uneven cutting. A wider strip has less problem. Based on our testing experience, a 5-mm wide strip from rabbit skin has much less variation. We thus made a mold specifically for this purpose. It used a piece of 1 mm-thick aluminum sheet. Five 50-mml length straight parallel cutting-through lines were made. The distance between each line is 5 mm (Fig. 1). When a skin is prepared, just place the mold on the skin, using a surgical knife to cut through each line, resulting in 4 same sized skin strips (30 mm long and 5 mm wide) for biomechanical testing.

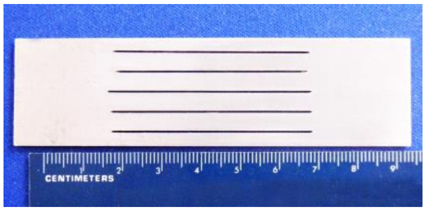

Figure S1. The aluminum skin strip mold. It can cut the skin to get 4 strips of 30-mm length and 5-mm width uniform rabbit skin strips.
